# Smokers′ Behavior and Perceptions in the Face of Increased Availability of Tobacco Harm Reduction Products in Pakistan: A Cross‐Sectional Survey in Islamabad and Rawalpindi

**DOI:** 10.1155/bmri/3735027

**Published:** 2026-02-27

**Authors:** Abdul Hameed, Daud Malik

**Affiliations:** ^1^ Department of Research and Development, Alternative Research Initiative, Islamabad, Pakistan

**Keywords:** combustible cigarettes, e-cigarettes, Pakistan, smoking cessation, tobacco control, tobacco harm reduction

## Abstract

The global tobacco crisis claims between 8 million lives annually with most of the 1.3 billion tobacco smokers being from low‐ and middle‐income countries, further emphasizing health disparities. Pakistan has a high burden of tobacco‐related illnesses, and it is among the 15 countries. Nonetheless, no national survey has been conducted in Pakistan to monitor the use of tobacco among adults since 2014 to measure tobacco reduction efforts. As a response to the harm reduction demand across the country, the cross‐sectional survey was carried out on 405 adult smokers within Islamabad and Rawalpindi. Data was collected using a structured and validated questionnaire. Descriptive and inferential statistical analysis was done using chi‐square, Fisher′s exact test, and logistic regression, respectively. The research identifies the essential variables affecting smoking cessation and adoption of THR, the importance of such demographic factors as age, education, and smoking intensity. The age factor was very significant since every extra year was combined with a 6.2% reduction of the probability of using THR (*β* = −0.062, 95% CI: −0.091 to −0.032, *p* < 0.001). Education was also positively influential in such a way that every next higher degree of education would raise the likelihood of using THR by 8.7% (*β* = 0.087, 95% CI: −0.013 to 0.161, *p* < 0.002). The employment status had a slight, but significant impact with the employed status raising the chances of using THR product usage by 43.3% (*β* = 0.433, 95% CI: −0.069 to 0.935, *p* < 0.009). The cigarette per day consumption became a significant predictor as an extra cigarette smoked raised the chances of using THR product by 6.8% (*β* = 0.068, 95% CI: −0.030 to 0.105, *p* < 0.001). Yet, one of the most significant information deficiencies between users of THR products is the reason why they often relapse into the habit of smoking once again. There is no difference in the age groups in terms of the perception of the safety of THR products, which makes it necessary to implement even more complex educational and awareness programs. Although age, education, and the intensity of smoking are all predictors of THR use and adoption, smoking duration and THR adoption showed no statistically significant correlation. On the whole, the paper is valuable in understanding the factors that will contribute to smoking cessation and adoption of harm reduction products in Pakistan.

## 1. Introduction

Globally, the tobacco pandemic takes over 8 million lives annually. The vast majority of 1.3 billion tobacco users in the world belong to low‐ and middle‐income countries (LMICs), where the consequences of smoking‐related diseases and deaths are enormous and contribute to health disparities [1, 2]. In developed countries, areas of comprehensive tobacco control have been put in place, leading to a reduction in smoking rates since the 1970s. This can be attributed to the increased awareness of the people on the need to focus on health, with the introduction of smoke‐free indoor areas and the introduction of spacing mechanisms such as pricing, legislation, and taxation [2]. In any case, the total number of smokers is on the rise due to the rise in the population of the world [3].

Smoking remains one of the major preventable causes of many health issues including liver, oral, and throat cancers; chronic obstructive pulmonary disease (COPD); heart disease; and stroke, particularly in countries such as India, Bangladesh, and Pakistan [4]. Pakistan is one of the top 15 countries with a high burden of tobacco‐related diseases. There was no national survey on a massive scale done on adult tobacco use in Pakistan since 2014 to enable a systematic monitoring of adult tobacco use and the tracking of key tobacco control indicators. An internationally recognized standard survey, Global Adult Tobacco Survey (GATS) was conducted in 2014, and in 2018, its metadata was published. The government of Pakistan and donor agencies consider this survey to have been nationally representative, which involved 9856 households. Out of these, 7831 individual interviews were filled in yielding a total response rate of 81.0% [5]. The survey showed that 31.8% of all adult men, 5.8% of all adult women, and 19.1% of all adults (equivalent to 23.9 million) were current tobacco users. In particular, 22.2% of males, 2.1% of females, and 12.4% of the total adult population (15.6 million) were current smokers [5].

Nevertheless, a recent study has determined Pakistan as a high‐burden country in terms of tobacco use whereby 31 million adults currently consume tobacco. According to a series of estimations done by the 2018–2019 Household Integrated Economic Survey (HIES) by Pakistan, it was found that tobacco was consumed by more than 45% of households. This ratio is a little bit different among various socioeconomic groups: 49% poor households and 38% wealthy households [6]. The tobacco control law in Pakistan is primarily the Prohibition of Smoking and Protection of Non‐smokers Health Ordinance 2002 that was further reinforced as Pakistan ratified the WHO Framework Convention on Tobacco Control (FCTC). There are a number of powerful actions that have been taken under this framework. These are 85% pictorial health warnings on cigarette packs on either side, prohibition of smoking at public places and transport means, and prohibition of sale of cigarettes to underage children. Signs of no smoking at the public places are also a requirement.

Moreover, the tobacco advertising in print and electronic media such as billboards, posters, and banners around the shops or mobile stalls is also prohibited completely. It has also prohibited the importation of shisha (in both tobacco and nontobacco) and associated products and the manufacture, importation or sale of cigarette packs, which have less than 20 cigarettes. Pakistan has raised the federal excise duty on locally made cigarettes. In 2023–2024, the lower and higher tiers had an increased share of the duty in the retail price by 48% and 68%. The increment of the duty to raise the costs of cigarettes is discussed as the win–win solution as far as the health and financial benefits of the state are concerned.

Nonetheless, the effectiveness of the tax increase has been challenged because of smuggling and illegal cigarettes in Pakistan. The tobacco control organizations and tobacco industry differ on the amount of smuggled and illegal cigarettes [7]. The use of tobacco is still the number one preventable cause of deaths in Pakistan. Each year tobacco causes about 160,100 deaths. Tobacco contributes to 66.5% of all deaths related to tracheal, bronchus, and lung cancer, 53.2% of all deaths caused by COPD, 21.9% of all deaths caused by ischemic heart disease, 15.2% of all deaths caused by diabetes mellitus, and 16.8% of all deaths caused by stroke [8]. In 2019, the total cost of all diseases and deaths associated with smoking reached Rs. 615.07 billion ($3.85 billion), with 70% of the total spending consisting of indirect costs (morbidity and mortality). Rural residents shoulder 61% of the total cost, with 77% incurred by men and 86% by persons aged 35–64 years [9].

The world is considering the idea of harm reduction to overcome the challenges of cessation of smoking and reduce some of the negative impacts of combustible smoking. Science, technology, and advancement of regulations have offered means of reducing the negative impact of potentially harmful activities. Harm reduction is one strategy where we recognize that the risk of harm may not have been fully removed but it is still better than the harm we were initially doing. An example of this is electric vehicles, which convey passengers between Point A and Point B just like their predecessors that used gasoline, but they emit significantly less pollution during the same journey. Similarly, instead of the total abstinence of nicotine, tobacco harm reduction (THR) can serve to diminish or eliminate the damage caused by the toxins generated during tobacco burning to individuals who cannot or will not stop smoking [10].

It can be used with four primary products, including snus (also called smokeless tobacco), e‐cigarettes (also called vapor products), heated tobacco products (also called heat‐not‐burn products), and nicotine replacement therapies (also called pouches, gums, and lozenges). Although THR proposes a less dangerous alternative to adult smokers, such risks as dual use, uptake by young people, and variability of product quality should undergo relevant regulation [11].

However, there is mistrust in the THR approach. This mistrust is based on the suspicion that the THR approach is driven by the tobacco industry. This mistrust is widespread in the form of misinformation and disinformation from tobacco control institutions, which are focusing on nicotine use and smoking. This misinformation has led to a split over the role of THR products in smoking cessation, adult consumers′ continued nicotine use, and concerns that safer nicotine products pose a risk to young people. However, at the same time, globally more than a billion adult smokers continue to use combustible cigarettes, posing serious risks to their health.

Effective harm reduction interventions, at minimal cost to governments and health agencies, can end smoking within a generation. The alternative is a continuation of approaches that will continue to fail those most in need and the price will be counted in the millions of lives that could have otherwise been saved [12, 13].

### 1.1. Study Objectives

The main objectives of this study are as follows:

• To analyze the behaviors and habits of smokers in Pakistan in the context of the increasing availability of THR products, such as e‐cigarettes.
• To assess smokers′ perceptions regarding the safety, effectiveness, and overall impact of THR products compared with traditional combustible cigarettes.
• To identify key factors that influence smokers′ decisions to quit smoking and adopt THR products.

### 1.2. Study Significance

The study provides valuable insights into the behavior and perceptions of smokers, which can help public health authorities develop more effective smoking cessation programs and policies that consider the potential role of THR products. Understanding the determinants that drive the adoption of THR products can inform policymakers in Pakistan about the potential benefits and risks associated with promoting these products as cessation tools. The findings can guide targeted educational campaigns to increase awareness about the relative safety and effectiveness of THR products, helping smokers make informed decisions. The study′s outcomes can be useful for stakeholders in the tobacco industry, including manufacturers and retailers of THR products, in understanding consumer behavior and tailoring their strategies to meet the needs of Pakistani smokers.

## 2. Materials and Methods

### 2.1. Data

Data from 405 participants gathered across the twin cities of Islamabad and Rawalpindi served as the basis for this analysis. The data were gathered using a well‐structured and validated questionnaire that covered a range of topics. These included demographic information such as age, education, employment status, and household income, along with smoking habits like duration, daily cigarette consumption, quit attempts, and methods used for quitting. Additionally, the questionnaire assessed respondents′ awareness and perceptions of THR products, including their awareness levels, sources of information, usage patterns, perceived safety and effectiveness, and factors influencing the adoption of THR products. The sample was randomly selected from the already existing list of 15,000 smokers, and data were collected through an online survey using telephone interviews (see Supporting Information “Study Questionnaire”).

## 3. Methods

### 3.1. Development of Questionnaire

Using literature and desk research, we developed a comprehensive, well‐structured quantitative survey questionnaire that was used to collect the data from Islamabad and Rawalpindi (see Supporting Information “Study Questionnaire” (available here)).

### 3.2. Data Collection and Sampling

To determine the optimal sample size for an empirical study, the size of the target population, the variance, the margin of error, and the desired level of confidence must be taken into consideration. When information about the standard deviation of variables is not available, the formula given below can be used to estimate the sample size. In this study, the sample size was estimated using the standardized Cochran formula.

(1) n0=Z2p1−pe2,

where *n*_0_ is the required sample size, expressed as the number of respondents. *Z* is a factor to achieve the 95% level of confidence. *p* is the predicted or anticipated value of the indicator, expressed in the form of a proportion (that 50% value was drawn from the optimal sample). *e* is margin of error (5%).

(2) n0=1.962.510.5−0.052=384.

The sample size of 384 was adjusted to 405 to account for approximately 5% nonresponse. It was based on acceptable norms and standards with a 5% margin of error, 95% confidence level, and 50% predicted or anticipated value of the indicator. The estimated sample size was almost equally distributed between the two cities, with 205 respondents from Rawalpindi and 200 from Islamabad.

### 3.3. Statistical Analysis

This study employed descriptive and inferential analyses, utilizing frequency and cross‐tabulation methods alongside chi‐square tests, Fisher′s exact test, and logistic regression to identify key factors influencing smoking cessation and the adoption of THR products. Specifically, for the logistic regression analysis, logit regression estimated these key factors. In practice, the choice between logit and probit often leads to similar results because the estimated probabilities from both models are quite close. Logit is generally more commonly used due to its simplicity and ease of interpretation [14]. This study employed two separate logistic regression analyses to examine factors influencing the cessation of combustible smoking and the adoption of THR products. The independent variables were smoker age, daily consumption, education level, employment status, household monthly income, number of smoking years, attempts to quit, and perceptions. The independent variables—age (in years), education (in years), employment status (dummy), household income (in PKR), consumption (in numbers), and perception (on a scale)—were used in their original form with few additional coding applied for education and perception.

The general form of the logit model used for analyzing quitting and adopting THR products is shown below:

(3) logitP=lnP1−P=β0+β1age+β2education+β3employment+β4household income+β5consumption+β6perception,

where *P* is the probability of the outcome occurring (e.g., the probability of quitting smoking or adopting THR), *P*/1 − *P* is the odds of the outcome, *l* *o* *g* *i* *t*(*P*) is the log‐odds of the outcome, *β*_0_ is the intercept of the model, and *β*_1_, *β*_2_ ⋯ ..*β*_7_ are the coefficients of the predictor variables.

## 4. Results

### 4.1. Smoker Demographics and Perceptions

Table 1 shows how different demographic variables such as age, education, occupation, and household income relate with the use of THR products. The median age of respondents aged 18–35 years is more likely to utilize THR products than the older ages, with the *p* value being significant as an indication of a strong relationship between age and use of THR products. Likewise, the level of education has an effect on consumption of THR products; well‐educated people with higher secondary education or higher education are more willing to consume these products than those with lesser education. The chi‐square *p* values of both age and education indicate that these variables have a great impact on an individual selecting THR products. The job and family income are essential in the consumption of a THR product. The highest rate of usage of THR products is observed among students; the employed and self‐employed ones being less inclined to use THR products. The *p* value indicates that there is a significant relation between profession and the use of THR. The same applies to the household income, whereby those in higher income households are more likely to use THR products than those in lower income households. The statistically significant *p* value attached to income suggests that economic factors play a significant role in the decision to use THR products and brings out socioeconomic inequality in access or choice of treatment.

**Table 1:** Smoker demographics and use of THR products.

		*n* (%)	THR Use		Chi‐square *p* value
			No (%)	Yes (%)	
Age	18–25	39.0	68.4	31.7	0.001
	26–35	28.4	74.8	25.2	
	36–45	19.8	87.5	12.5	
	Above 45	12.8	92.3	7.7	
Education	Illiterate	7.9	87.5	12.5	0.011
	Primary	17.0	85.5	14.5	
	Secondary	25.7	82.7	17.3	
	Higher secondary	26.4	69.2	30.8	
	BA or master or higher	23.0	69.9	30.1	
Occupation	Employed	58.0	80.4	19.6	0.001
	Self‐employed	28.6	82.8	17.2	
	Unemployed	6.4	57.7	42.3	
	Student	6.9	42.9	57.1	
Household monthly income	Below 20,000 PKR	2.2	66.7	33.3	0.001
	20,000–40,000 PKR	24.9	91.1	8.9	
	40,001–60,000 PKR	22.7	79.4	20.7	
	Above 60,000 PKR	50.1	69.5	30.5	

*Source*: Authors′ estimations.

Table 2 shows the information on the perceptions of the safety of THR products among different age groups, education levels, occupations, and household incomes. Perceptions of the participants are divided into four groups, namely, much safe, somewhat safe, equally safe, and less safe as compared with combustible smoking. The difference in perception between age groups is not very large, which is demonstrated by the chi‐square *p* value of 0.875. Nonetheless, the older age groups especially over the age of 45 perceive THR products as either much safe or less safe without any in between. On the contrary, younger people, in particular, people aged between 18 and 25 years have a more diversified view, yet most of them believe that THR products are “less safe” too. Table 2 also shows the findings on the perception of respondents to the use of THR products all alternatives used by the respondents with regard to their age, education, and other parameters.

**Table 2:** Smoker demographics and perception about THR.

		Perception about THR				Chi‐square *p* value
		Much safe	Somewhat safe	Equally safe	Less safe	
Age	18–25	28.0	12.0	8.0	52.0	0.875
	26–35	34.5	13.8	10.3	41.4	
	36–45	10.0	10.0	10.0	70.0	
	Above 45	50.0	0.0	0.0	50.0	
Education	Illiterate	0.0	0.0	25.0	75.0	0.398
	Primary	30.0	10.0	20.0	40.0	
	Secondary	38.9	11.1	0.0	50.0	
	Higher secondary	36.4	6.1	6.1	51.5	
	BA or master or higher	17.9	21.4	10.7	50.0	
Occupation	Employed	23.9	17.4	8.7	50.0	0.728
	Self‐employed	30.0	5.0	15.0	50.0	
	Unemployed	45.5	9.1	0.0	45.5	
	Student	31.3	6.3	6.3	56.3	
Household monthly income	Below 20,000 PKR	0.0	33.3	33.3	33.3	0.120
	20,000–40,000 PKR	33.3	0.0	33.3	33.3	
	40,001–60,000 PKR	26.3	10.5	5.3	57.9	
	Above 60,000 PKR	30.7	12.9	4.8	51.6	

*Source*: Authors′ estimations.

The questions related to perception were meant to determine whether the respondents believe that THR products are as healthy to use as combustible cigarettes. In general, the opinions concerning THR and combustible smoking are still unclear, and the level of knowledge regarding THR is low. A significant portion of the users believe that there is not so much difference between smoking and THR products with regard to harm.

The education influence of the perception of THR reveals that people who are better educated (bachelors or master) tend to be more diverse on their perception and some people think of them as being somewhat safe, others as being less safe. Less educated people and those who are illiterate, mostly perceive THR products as being less safe whereas the chi‐square *p* value of 0.398 indicates that there is no significant relationship between the perception and education. Perception is also subject to occupation and income. As an example, students and unemployed people perceive THR products as being less safe, whereas employed or self‐employed people have a more balanced perception. The perceptions of the lower income groups (less than 20,000 PKR) are different, yet the *p* value of 0.120 shows that the differences are not significant (Table 2).

Table 3 provides the information about the perceptions of the various demographic groups regarding the perception of THR products as a cessation tool. The results are categorized into those ones who have thought of THR as a cessation product (yes) and those who do not (no). Concerning age, people (18–35) are more inclined to use THR products as a cessation tool (54% and 44%) than the older age groups. Persons beyond 45 years are the least likely (25%) to regard THR products as cessation tool. Nevertheless, the *p* value of chi‐square is 0.586, which shows that the difference between age groups is not statistically significant. The education level also demonstrates some discrepancy with the higher secondary education (63.6%) and illiterates (50%) showing a higher probability of viewing THR products as a cessation tool than those having bachelor or master degree (35.7%). However, the *p* value of 0.261 shows that there is no significant statistical correlation between education and the perception of THR products as a cessation tool. Students when analyzing occupation will most likely perceive THR products as cessation tools (56.3%), with employed, self‐employed, and unemployed people having an even distribution with slight majority not regarding THR products as cessation tools. The *p* value of 0.912 indicates that occupation is not a significant factor in causing this perception. Concerning the household income, people with the poorest income (less than 20,000 PKR) are most likely to see THR as cessation tool (66.7%), just like those in the income range of 20,000–40,000 PKR. It is however less inclined among those in the highest income bracket (above 60,000 PKR), of which it is only 45.2%. Although there are these differences, the chi‐square *p* value of 0.599 indicates that the household income is not affected statistically on whether individuals regard THR products as cessation tool.

**Table 3:** Smoker demographics and THR products as a cessation tool.

		Consider THR as a cessation tool		Chi‐square *p* value
		No	Yes	
Age	18–25	46.0	54.0	0.586
	26–35	55.2	44.8	
	36–45	60.0	40.0	
	Above 45	75.0	25.0	
Education	Illiterate	50.0	50.0	0.261
	Primary	60.0	40.0	
	Secondary	55.6	44.4	
	Higher secondary	36.4	63.6	
	BA or master or higher	64.3	35.7	
Occupation	Employed	52.2	47.8	0.912
	Self‐employed	55.0	45.0	
	Unemployed	54.6	45.5	
	Student	43.8	56.3	
Household monthly income	Below 20,000 PKR	33.3	66.7	0.599
	20,000–40,000 PKR	33.3	66.7	
	40,001–60,000 PKR	52.6	47.4	
	Above 60,000 PKR	54.8	45.2	

*Source*: Authors′ estimations.

Table 4 provides the demographics of smokers and the way they would want to quit smoking. This paper classifies all the THR strategies that include e‐cigarettes, smokeless tobacco, nicotine pouches, and NRTs as alternative categories. Doctor‐recommended prescription medicines are combined under the prescription medications, whereas willpower, exercise, and therapies fall under the willpower strategies. The others category involves traditional forms, which include candy, bubbles, and niswar. The majority of the smokers claimed that the most significant strategies to quit smoking combustibles are those that rely on the force of will. Smokers between 18 and 35 years claimed that alternative means, like vaping or nicotine replacement, are the best of all in quitting combustible smoking. On the same note, smokers who had a higher education, students, and those who had a higher income bracket also thought that alternatives were the best method of quitting. A few smokers felt that prescription drugs were better, although this was not a statistically significant group. The Fisher′s exact test has been utilized in this table because some of the cells have the expected counts of fewer than five.

**Table 4:** Smoker demographics and methods to quit smoking.

		Willpower strategies	Alternatives	Prescription medications	Others	Fisher′s exact *p* value
Age	18–25	40.2	59.8	0.0	0.0	0.001
	26–35	47.5	52.5	0.0	0.0	
	36–45	62.0	34.0	0.0	4.0	
	Above 45	66.7	23.1	5.1	5.1	
Education	Illiterate	69.6	21.7	4.4	4.4	0.001
	Primary	56.4	41.0	0.0	2.6	
	Secondary	56.9	38.5	1.5	3.1	
	Higher secondary	40.6	59.4	0.0	0.0	
	BA or master or higher	45.0	55.0	0.0	0.0	
Occupation	Employed	52.8	43.8	0.7	2.8	0.033
	Self‐employed	59.2	40.9	0.0	0.0	
	Unemployed	23.8	71.4	4.8	0.0	
	Student	35.0	65.0	0.0	0.0	
Household monthly income	Below 20,000 PKR	55.6	44.4	0.0	0.0	0.092
	20,000–40,000 PKR	58.2	35.8	1.5	4.5	
	40,001–60,000 PKR	50.0	46.3	1.9	1.9	
	Above 60,000 PKR	46.8	53.2	0.0	0.0	

*Source*: Authors′ estimations.

### 4.2. Smoking Habits and Perceptions

Table 5 includes the information about the correlation between smoking habits (including the period of smoking and the number of smoking cigarettes a day) and smoking THR products. Regarding the duration of smoking, people with 6–10 years of smoking are the most inclined to use THR products, and 37.5% of them use it. The respondents with a smoking history less than 1 year and over 10 years showed a slightly lower use of THR products. Nevertheless, the chi‐square *p* value of 0.326 is not significant enough to indicate that there is a statistically significant relationship between the length of smoking and the use of THR products. Concerning the cigarettes per day, the highest rate of THR product consumption is in the group of smokers who smoke more than 20 cigarettes per day, and the lowest rate is in the group of smokers who smoke less than five cigarettes per day (43.8% and 18.2%, respectively). The *p* value of 0.078 represents a value near the statistical significance of the number of cigarettes smoked per day and its relationship with THR use.

**Table 5:** Smoking habits and use of THR products.

		*n* (%)	THR use		Chi‐square *p* value
			No (%)	Yes (%)	
Smoking time	Less than 1 year	6.2	76.0	24.0	0.326
	1–5 years	35.1	70.4	29.6	
	6–10 years	19.8	62.5	37.5	
	More than 10 years	39.0	73.4	26.6	
Number of cigarettes per day	< 5	10.9	81.8	18.2	0.078
	5–10	48.9	68.2	31.8	
	11–20	32.4	73.3	26.7	
	> 20	7.9	56.3	43.8	
Try to quit smoking	No	36.8	99.3	0.7	0.001
	Yes	63.2	53.5	46.5	
Number of times	One	15.6	45.0	55.0	0.335
	Two	19.9	62.8	37.3	
	Three	16.4	57.1	42.9	
	More than three	48.1	51.2	48.8	

*Source*: Author′s estimations.

The relationship between quitting smoking attempts and the use of THR products is also discussed in Table 5. Persons who have never attempted to quit smoking are extremely infrequent users of THR products with a shocking 99.3% that have never used it. On the other hand, individuals who have tried to quit smoking are significantly more likely to use THR products, and 46.5% of them said they did. The chi‐square *p* value of 0.001 indicates a strong and statistically significant correlation between the attempts to quit smoking and the use of THR products. Considering the number of quit attempts, the relationship between the attempt only once is most likely to use THR products (55%), but the relation is not found to be statistically significant because the *p* value of this case is 0.335.

Table 6 demonstrates that the perceptions of the safety of THR products differ depending on different smoking behaviours. Smokers who have smoked various durations tend to have a similar view of THR with approximately half of each group viewing it as “much safe.” As an illustration, smokers who have less than 1 year and more than 10 years of smoking experience 50% of them as THR products as much safe, and a large proportion of them as less safe. Nevertheless, the *p* value of the chi‐square of 0.77 indicates that there is no significant statistical significance between the smoking duration and the perception of the safety of THR products. In the same way, at the number of daily cigarette smokers, individuals who smoke fewer than 5 and more than 20 cigarettes daily are more likely to consider THR products as much safe, but the *p* value of 0.58 was found to be not statistically significant.

**Table 6:** Smoking habits and perception of THR products.

		Perception about THR				Chi‐square *p* value
		Much safe	Somewhat safe	Equally safe	Less safe	
Smoking time	Less than 1 year	50.0	0.0	16.7	33.3	0.773
	1–5 years	50.0	11.9	9.5	28.6	
	6–10 years	43.3	20.0	10.0	26.7	
	More than 10 years	50.0	7.1	4.8	38.1	
Number of cigarettes per day	< 5	62.5	0.0	0.0	37.5	0.582
	5–10	46.0	15.9	11.1	27.0	
	11–20	42.9	8.6	8.6	40.0	
	> 20	64.3	7.1	0.0	28.6	
Try to quit smoking	No	0.0	0.0	0.0	100.0	0.537
	Yes	48.7	11.8	8.4	31.1	
Number of times	One	45.5	13.6	9.1	31.8	0.422
	Two	47.4	15.8	5.3	31.6	
	Three	72.2	16.7	0.0	11.1	
	More than three	43.3	8.3	11.7	36.7	

*Source*: Authors′ estimations.

Table 6 also examines the effect of the efforts to quit smoking on the perception of the safety of THR products. Remarkably, the strongest skepticism is in the category are those who have never tried to quit smoking and, in that case, regard THR products as the less safe (100%). On the other hand, the individuals who have tried to quit smoking are in a better perception towards THR products and almost half of the people thought that they are safe to a much larger extent. There are some differences in the perceptions of those who have made several attempts to quit with a significant figure of 72.2% who have attempted three times perceiving THR products as much safe. The chi‐square *p* values (between 0.422 and 0.537) indicate, however, that the relationship between quitting attempts and THR perception is not statistically significant.

Table 7 indicates that the perception of smokers towards THR products as smoking cessation tools is dependent on the length of time and the number of cigarette packs that the person smokes on a daily basis. The perception of the individuals who have been smoking varied lengths of time tends to be quite similar, with a slight majority in each group believing that THR products are a cessation tool. The less than 1‐year smokers (66.7%) and over 10‐year smokers (61.9%) show THR products as a cessation aid. The chi‐square *p* value of 0.698 is, however, indicating that there is no statistically significant correlation between the perception of THR products as cessation aids and smoking duration. Regarding the quantity of cigarettes smoked daily, smokers who smoke less than 5 cigarettes tend to the greatest extent to adopt THR products as a smoking‐quitting tool (87.5%), compared with smokers who smoke 11–20 or more than 20 cigarettes daily (57.1% and 57.1%, respectively). Nonetheless, this tendency, although, the *p* value of 0.149 shows that the relationship is not statistically significant.

**Table 7:** Smoking habits and THR as a cessation tool.

		Consider THR as a cessation tool		Chi‐square *p* value
		No (%)	Yes (%)	
Smoking time	Less than 1 year	33.3	66.7	0.698
	1–5 years	26.2	73.8	
	6–10 years	30.0	70.0	
	More than 10 years	38.1	61.9	
Number of cigarettes per day	< 5	12.5	87.5	0.149
	5–10	25.4	74.6	
	11–20	42.9	57.1	
	> 20	42.9	57.1	
Try to quit smoking	No	100.0	0.0	0.140
	Yes	31.1	68.9	
Number of times	One	40.9	59.1	0.075
	Two	47.4	52.6	
	Three	11.1	88.9	
	More than three	28.3	71.7	

*Source*: Authors′ estimations.

### 4.3. Methods Used for Quitting and THR

**Figure 1:**
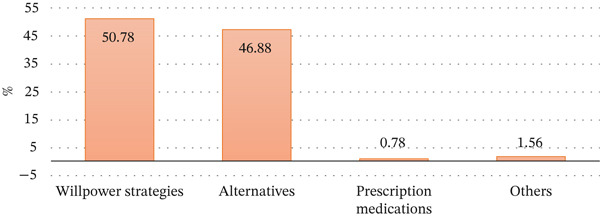
Method used for quitting.

Figure 1 gives a demonstration of the preferences of various strategies to be used in achieving smoking cessation. Most people (50.78%) are using willpower strategies as their main strategy, which means that they prefer using self‐discipline. Right after that, 46.88% of the respondents preferred other approaches (THR) including nicotine replacement therapy or other over‐the‐counter assistance. A very low proportion (0.78%) of people used prescription medication, and 1.56% chose the other category, which comprised traditional medication like candy or toffees, which can also comprise less common and unspecified options. The majority of smokers do not want to use medical solutions; they only use personal willpower or other options instead of prescribed drugs.

**Figure 2:**
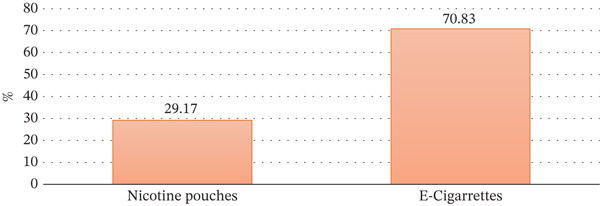
Use of THR.

The percentage usage of nicotine pouches against e‐cigarettes is shown in Figure 2. E‐cigarettes have an enormously higher level of usage (70.83%) than nicotine pouches whose usage is 29.17%. The difference in visual representation of the bar highlights the obvious difference in preference for the two methods of nicotine delivery.

**Figure 3:**
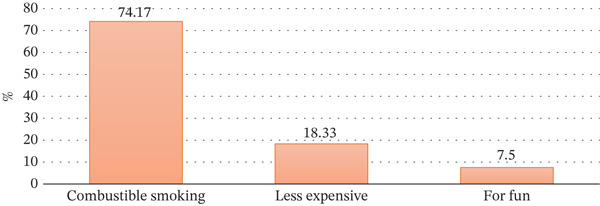
Intent of using the smoking cessation product.

Figure 3 presents the motives of using combustible smoking among respondents that will be divided into three groups, which are “combustible smoking” and “less expensive and fun.” Most smokers (74.17%) smoke out of love, which is a high preference or addiction. Cost is a major factor in influencing the choice of smoking because only 18.33% of smokers choose to smoke because it is cheaper. Finally, the reasons that are presented have also shown that recreational use is the least recognized source of motivational smoking; only 7.5% of the respondents are found to be doing it for fun. This proves that the preferences of smoking are the leading determinants of smoking behavior as opposed to price or pleasure.

### 4.4. Key Factors of Quitting Smoking and Using THR as Smoking Cessation Products

Table 8 presents the outcome of the logistic regression of the most important factors of the application of THR products as a tool of smoking cessation. The overall model fit is displayed that the likelihood ratio chi‐square statistic of 40.84 adhering to 6 degrees of freedom demonstrates the overall model fit was significantly better than a model with no predictors (i.e., the null model). The chi‐square test value has a *p* value of less than the 0.0001, which shows that the model is statistically significant in general. The figure is the ratio of the variation that is explained by the model. The value of 0.1155 shows that the model explains around 11.5% of the variation on the dependent variable (use of THR products). The age coefficient is approximated to be −0.062. The log‐odds of using THR products reduces by 0.062 with an increase in age by one unit (year), other things remaining constant. The *p* value, which is less than 0.00, shows that the effect of age is significant at the 1% mark. The age effect is significant, and 0 is not within the 95% confidence interval (−0.091 and −0.032). The coefficient of education indicates that with every increment of 1 unit in the level of education (e.g., years of additional education) an incremental increase of 0.087 log‐odds of using THR products, all other factors being equal. Education has a statistically significant effect of 5% level. The confidence interval excludes a 0, which proves the level of significance of education effect. Employment raises the log‐odds of THR products use by 0.433, relative to unemployment. The level of employment status is slightly significant at 10% level. There is no significant impact of household income on the log‐odds of THR use. The log‐odds of using THR products are 0.068 greater with an increase in the number of cigarettes smoked in a day. The influence of cigarette use has a statistically significant level of 1%. The variables that are of significant predictors include age, education, and consumption of cigarettes per day as the smoking cessation tool of THR products. Nevertheless, because the analysis is conducted on the one‐point in time data, the threat of reverse causality must be considered, as individuals who utilize THR products may become more aware of their positive attitude to them after adoption and not prior to it.

**Table 8:** Key factors of adopting THR.

*N* *u* *m* *b* *e* *r* of *o* *b* *s* = 256						
*L* *R* *c* *h* *i*2(6) = 40.84						
*P* *r* *o* *b* > *c* *h* *i* ^2^ = 0.0000						
*P* *s* *e* *u* *d* *o* *R* ^2^ = 0.1155						
*L* *o* *g* *l* *i* *k* *e* *l* *i* *h* *o* *o* *d* = −156.39						
Use of THR (yes/no)	Coefficient	Std. err.	*z*	*p* > *z*	95% conf. interval	
Age	−0.062	0.015	−4.080	0.001	−0.091	−0.032
Education	0.087	0.038	2.320	0.020	0.013	0.161
Employment status	0.433	0.256	1.690	0.090	−0.069	0.935
Household income	0.000	0.000	0.010	0.990	0.000	0.000
Cigarettes consumption per day	0.068	0.019	3.530	0.001	0.030	0.105
Constant	−0.580	0.992	−0.590	0.560	−2.524	1.363

*Source*: Authors′ estimations.

The major factors of attempt to quit smoking are also presented in Table 9. The likelihood ratio chi‐square test value in Model 2 is 141.42 on 6 degrees of freedom representing that the entire model fits significantly better than a model with no predictors (i.e., the null model). The chi‐square statistic has a *p* value of less than black, meaning that the model is statistically significant. A value of Pseudo *R* ^2^ is 0.2701. It means that the model explains about 27.01% of the variation of the dependent variable (tried to quit smoking). The log‐odds of attempting to quit smoking also rises by 0.046 with an increase in age by one unit (year), other factors being held constant. The *p* value shows that the age effect has a statistical significance of 1%. There is no statistically significant correlation between the effect of education, employment, household income, and cigarette consumption on an attempt to quit smoking. It is interesting that the perception of the usage and attempt to quit smoking are extremely correlated with the use of THR products. The log‐odds of attempting to quit smoking is raised by 2.050 by a positive perception of THR. The statistical significance of the perception of THR is significant at 1%. Nonetheless, because the data is cross‐sectional, the threat of reverse causality cannot be disregarded, that is, those people who tried to quit smoking might have later formed more positive attitudes towards THR products.

**Table 9:** Key factors of trying to quit smoking.

*N* *u* *m* *b* *e* *r* of *o* *b* *s* = 405						
*L* *R* *c* *h* *i*2(6) = 141.42						
*P* *r* *o* *b* > *c* *h* *i* ^2^ = 0.0000						
*P* *s* *e* *u* *d* *o* *R* ^2^ = 0.2701						
*L* *o* *g* *l* *i* *k* *e* *l* *i* *h* *o* *o* *d* = −191.049						
Tried to quit smoking (yes/no)	Coefficient	Std. err.	*z*	*p* > *z*	95% conf. interval	
Age	0.046	0.012	3.860	0.001	0.023	0.070
Education	−0.012	0.031	−0.390	0.696	−0.073	0.049
Employment status	0.279	0.256	1.090	0.277	−0.224	0.782
Household income	0.000	0.000	−1.070	0.284	0.000	0.000
Cigarettes consumption per day	−0.020	0.016	−1.300	0.193	−0.051	0.010
Perception of THR	2.050	0.493	4.150	0.001	1.083	3.017
Constant	−1.015	0.753	−1.350	0.178	−2.491	0.462

*Source*: Authors′ estimations.

## 5. Discussions

The findings give a detailed picture on the relationship between different demographic variables, smoking patterns, perceptions, adoption, and perception of THR products. The findings indicate that there are important relationships between some demographic features and the use of the THR products, as well as the perception of the use of the THR as a cessation tool and the safety thereof. The results show that people (especially young) are more inclined to use THR products than older age groups. This is similar to larger tendencies among younger populations that demonstrate greater tolerance towards other nicotine products. In Pakistan, recent national statistics on tobacco and combustible cigarette smoking do not exist, and the last GATS has been undertaken in 2014 [4, 15]. The average age at which smoking was initiated in the daily smokers aged 20–34 years was 18.7 years, according to GATS [16].

The Pakistan Demographic and Health Survey (2012–2013) gave a smoking prevalence rate of 46% of the individuals aged 15–39 years [17] as compared with the 39% in the National Health Survey 1990–1994 [18]. All these findings support the necessity to revise surveillance and study of smoking behavior and tobacco harm reduction in Pakistan. In Pakistan, the major factor that affects the brand of cigarette smoked is affordability. The price of the smoked product is highly sensitive, and due to this, smokers tend to move to local brands, which are relatively cheap. The fact that the age group of 18–35 years adopts the THR products more might indicate experimentation and not informed substitution because most users do not have sufficient medical and scientific knowledge regarding THR. Such an inconsistency puts the risk of dual use (smoking cigarettes and THR products) or returning to combustible cigarettes. Thus, the use of policy should be aimed at proper regulation so that THR products could be available to adult smokers but prevent their use by never‐smokers and young people.

Pakistan still has a low awareness and availability of cessation services. Not every person knows that he or she can receive medical help to quit smoking. Furthermore, the myths concerning nicotine and THR products are still there. Most of the Pakistani doctors (70%) think that nicotine causes cancer [4]. In another systematic review, the inconsistency of tobacco nicotine education and training to healthcare providers had been reported [19]. The proposed strategies to promote THR and adopt evidence‐based cessation techniques include strengthening professional education and introducing valid nicotine science into medical education.

Another important factor related to THR adoption is education level. The more educated a person, the greater is the tendency to take THR products, implying that access to knowledge and health literacy determines behavior. Although age and education have a very strong impact on the usage of THR, there was no significant difference in perception of safety between age groups. This indicates that demographics are not as important in influencing knowledge about attitudes towards THR safety as general awareness and social context.

The statistical insignificance of differences displays the necessity to conduct extensive health communication campaigns to enhance the knowledge about THR products and their comparative risks. The correlation between the smoking habits and the use of THR provides additional information. Even though the smoking time was not significantly connected with using THR, the number of smoked cigarettes per day was quite close to significance, which proves that smokers who smoke more cigarettes daily might be more willing to use THR. Also, those who had made efforts to quit were much more likely to report the use of THR products, implying that the use of THRs is linked to quit attempts.

Nevertheless, due to the cross‐sectional design, no causality can be made out of it, as people with positive attitudes toward THR can be more willing to quit, or those who have tried to quit can form more positive attitudes toward THR. The logistic regression model was able to identify important predictors of THR adoption and quit attempts. Significant predictors of THR use included age, education, and cigarette use, whereas there was no significant relationship between employment and income.

Similarly, when there was a positive perception of THR, it was strongly associated with increased chances of quit attempts. These correlations indicate that perceptions can be very important in the development of cessation‐related behaviors, and that there is a need to have public health messages that are well‐targeted. Nevertheless, a number of limitations should be taken into consideration. To begin with, the study did not imply causal inference as a cross‐sectional design was taken. Second, the two cities had a small sample of data collection and this could not be generalized to the larger population. Third, smoking and THR behaviors were self‐reported, and no biochemical confirmation was done, which may cause recall or socially desirable bias. Fourth, observed associations could be confounded by some of the relevant variables that were not measured, including the level of nicotine dependence, stress, mental health, and tobacco control policy exposure.

Lastly, although THR products might promise advantages to the current smokers in terms of harm reduction, the market in LMICs such as Pakistan is not well‐regulated. Heterogeneity of products, the presence of different concentrations of nicotine, and quality inconsistency are a challenge to the users′ health. Besides, duality and relapse processes are typical, which highlights the fact that not all THR adoption translates into smoking cessation. As such, the policies must ensure a compromise between the possibility of reducing harm and high consumer protection guidelines and educating people to reduce unintended consequences.

Similar to the previous findings, quitting efforts and the intensity of smoking play a crucial role in determining the use of THR products. The most of respondents used e‐cigarettes as a THR product. A survey with a 1‐year follow‐up study indicates that daily e‐cigarette use, alongside smoking, is associated with increased rates of attempts to quit smoking and a reduction in smoking [20]. The longitudinal survey study found that daily vaping was linked to an increased likelihood of planning to quit smoking at follow‐up among individuals who initially had no plans to quit [21]. The literature also indicates that using e‐cigarettes with high nicotine concentration, compared with those with no nicotine, is associated with an increased number of cigarettes smoked per day at follow‐up [22].

## 6. Conclusions and Recommendations

The results provide a comprehensive understanding of how demographic factors, smoking behaviors, and perceptions are associated with the adoption and perception of THR products in Pakistan. The analysis highlights significant associations between demographic characteristics—particularly age, education, and smoking intensity—and the likelihood of using THR products. Individuals (aged 18–35) and those with higher education levels are more inclined to adopt THR products, reflecting broader trends in the acceptance of newer smoking alternatives. However, the study also reveals critical gaps in medical and scientific knowledge regarding THR products, especially among users, who are more likely to adopt these products without adequate understanding. This lack of knowledge often leads to a relapse into combustible smoking. Moreover, perceptions of THR safety do not significantly differ across age groups, suggesting a need for more consistent education and awareness efforts. The study underscores the importance of medical awareness and education for healthcare professionals, tobacco control advocates, and the general public. There is strong evidence supporting the need for widespread dissemination of accurate information about nicotine and THR products to address the misconceptions that hinder smoking cessation efforts.

• Implement targeted education and awareness campaigns to improve the understanding of THR products, particularly among younger (age 18–35) populations and healthcare professionals. These campaigns should focus on the safety, efficacy, and appropriate use of THR products as a smoking cessation tool. Launch targeted, evidence‐based campaigns to clarify the relative risks of nicotine, THR, and combustible tobacco, addressing common misconceptions that hinder cessation efforts.
• Proper regulations should be established to guide the use of THR products among current smokers while discouraging their use by nonsmokers and new users. Policymakers should consider age restrictions, product labeling, and marketing regulations to prevent the uptake of THR products by individuals without a prior smoking history. Integrate THR options within smoking cessation programs for adult smokers, while maintaining a strong focus on preventing initiation among nonsmokers and youth.
• Train pharmacists, physicians, and allied health professionals to deliver nicotine education, cessation counseling, and THR guidance based on scientific evidence. This would enable them to provide better guidance and support to smokers seeking to quit. Incorporate brief cessation advice and harm reduction counseling into routine consultations at primary care facilities.
• Smoking cessation programs should be strengthened with a focus on integrating THR products as a potential aid. These programs should be accessible and promoted widely, especially in regions with high smoking prevalence.
• Continued research is necessary to monitor the long‐term effects of THR products′ usage, particularly in different demographic groups. Future studies should also explore the impact of public health messaging on perceptions and behaviors related to THR.

## Author Contributions

All the authors made equal contributions to this study.

## Funding

This work was supported by the Global Action to End Smoking.

## Disclosure

The contents, selection, and presentation of facts, as well as any opinions expressed herein, are the sole responsibility of the authors and under no circumstances shall be regarded as reflecting the positions of the Global Action to End Smoking.

## Ethics Statement

This study was conducted in accordance with the ethical standards of the Alternative Research Initiative (ARI). Ethical approval was obtained from the ARI Research Committee. All participants were informed about the objectives of the study and provided verbal informed consent prior to participation. The confidentiality and anonymity of all participants were strictly maintained throughout the research process.

## Conflicts of Interest

The authors declare no conflicts of interest.

## Supporting information


**Supporting Information** Additional supporting information can be found online in the Supporting Information section. This questionnaire is designed to assess smokers′ demographic characteristics, smoking habits, and quit attempts, as well as their awareness, perceptions, and willingness to use tobacco harm reduction products in Pakistan. It also explores factors that may encourage or discourage the adoption of tobacco harm reduction products, along with participants′ concerns and opinions. The tool is aimed at generating evidence on smoking behavior and attitudes toward tobacco harm reduction to inform public health strategies and policy discussions.

## Data Availability

All relevant data are presented in the manuscript. The complete data set can be obtained from the corresponding author upon request.
