# Supplementary material for: Smokers′ Behavior and Perceptions in the Face of Increased Availability of Tobacco Harm Reduction Products in Pakistan: A Cross‐Sectional Survey in Islamabad and Rawalpindi
Source: Biomed Res Int. 2026 Feb 27;2026:3735027. doi: 10.1155/bmri/3735027 (PMC12949362; doi:10.1155/bmri/3735027)
Supplement: Supplementary file 1 — Supporting Information Additional supporting information can be found online in the Supporting Information section. This questionnaire is designed to assess smokers′ demographic characteristics, smoking habits, and quit attempts, as well as their awareness, perceptions, and willingness to use tobacco harm reduction products in Pakistan. It also explores factors that may encourage or discourage the adoption of tobacco harm reduction products, along with participants′ concerns and opinions. The tool is aimed at generating evidence on smoking behavior and attitudes toward tobacco harm reduction to inform public health strategies and policy discussions. [file BMRI-2026-3735027-s001.pdf]

A study on smokers' behaviour and perceptions in the face of increased availability of THR products in Pakistan

Section 1: Demographic Information

Age:

- 18-24
- 25-34
- 35-44
- 45-54
- 55 or above

Gender:

- Male
- Female

Educational Level:

- Less than high school
- High school graduate
- Some college/University
- Bachelor's degree
- Master's degree or higher

Occupation:

- Employed full-time
- Employed part-time
- Student
- Unemployed
- Other (please specify)

Monthly Household Income:

- Below 20,000 PKR
- 20,000 - 40,000 PKR
- 40,001 - 60,000 PKR
- Above 60,000 PKR

Section 2: Smoking Habits

How long have you been smoking?

- Less than 1 year
- 1-5 years

- 6-10 years
- More than 10 years

On average, how many cigarettes do you smoke per day?

Have you tried to quit smoking in the past?

- Yes
- No

If yes, what methods have you used to quit smoking? (Check all that apply)

- Nicotine replacement therapy (NRT)
- Prescription medications
- Cold turkey
- Counseling or support groups
- Other (please specify)

Section 3: Awareness and Perceptions of THR Products

Have you heard of Tobacco Harm Reduction (THR) products such as e-cigarettes, vaping devices, or heat-not-burn tobacco products?

- Yes
- No

If yes, what was the source of your information? (Check all that apply)

- Television
- Internet
- Social media
- Newspapers/magazines
- Healthcare providers
- Friends/family
- Other (please specify)

What are your perceptions regarding the safety and effectiveness of THR products compared to traditional cigarettes?

- Much safer
- Somewhat safer
- Equally safe
- Less safe
- Don't know

Would you consider using THR products as an alternative to smoking traditional cigarettes?

- Yes
- No
- Not sure

Section 4: Factors Influencing Adoption of THR Products

What factors would make you more likely to try or use THR products? (Check all that apply)

- Health benefits
- Cost-effectiveness
- Availability/accessibility
- Peer influence
- Marketing/advertising
- Government regulations
- Other (please specify)

What concerns or barriers do you have that would prevent you from trying or using THR products? (Check all that apply)

- Health risks associated with THR products
- Lack of information
- Social stigma
- Legal restrictions
- Cost
- Preference for traditional cigarettes
- Other (please specify)

Section 5: Additional Comments

Please share any additional thoughts or comments you have regarding Tobacco Harm Reduction products or smoking behavior in Pakistan.
